# Preventing the Risk of Dental Problems in Children With Special Health Care Needs: A Case Series

**DOI:** 10.7759/cureus.86320

**Published:** 2025-06-18

**Authors:** Varsha Sharma, Sushmita Pattnaik, Brahmananda Dutta, Priti Shukla, Birupakshya Mahakul, Kanika S Dhull

**Affiliations:** 1 Pediatric Dentistry, Maharshi Vashishtha Autonomous State Medical College, Basti, Rampur, IND; 2 Pediatric and Preventive Dentistry, Government Medical College and Hospital, Sundargarh, IND; 3 Pediatric and Preventive Dentistry, Department of Health and Family Welfare, Government of Odisha, Bhubaneswar, IND; 4 Pedodontics and Preventive Dentistry, Kalinga Institute of Dental Sciences (KIDS) Kalinga Institute of Industrial Technology (KIIT) (deemed to be university), Bhubaneswar, IND; 5 Orthodontics, All India Institute of Medical Sciences, Raebareli, Raebareli, IND; 6 Physiotherapy, Kalinga Institute of Medical Sciences (KIMS) and Hospital, Bhubaneswar, IND

**Keywords:** adhd, autism, cleft lip and palate, hearing impaired, social anxiety disorder, special health care dentistry

## Abstract

In India, a significant number of children are born with disabilities. These children are at higher risk of facing dental issues in contrast to healthy children. The oral health of children with special health care needs (SHCNs) usually deteriorates faster than that of the general population as they grow older. Poor oral health can result in nutritional intake deficiency, reduced social interactions, difficulty in undertaking day-to-day activities, and related anxiety. As per the American Academy of Pediatric Dentistry (AAPD) recommendations, preventing the risk of developing oral disease is a fundamental part of the comprehensive oral health care for children with special needs. The presented case series aims to promote preventive care for differently abled children, which can improve their quality of life.

## Introduction

The American Academy of Pediatric Dentistry (AAPD) defines special health care needs (SHCN) as “any physical, developmental, mental, sensory, behavioral, cognitive, or emotional impairment or limiting condition that requires medical management, health care intervention, and/or use of specialized services or programs. The condition may be congenital, developmental, or acquired through disease, trauma, or environmental cause and may impose limitations in performing daily self-maintenance activities or substantial limitations in a major life activity” [1]. Children with SHCN may include those with developmental (e.g., cerebral palsy) or cognitive (e.g., intellectual disability) disorders, behavioral (e.g., attention-deficit hyperactivity disorder, autism spectrum disorder, anxiety), congenital (e.g., trisomy 21, congenital heart disease), and systemic diseases (e.g., childhood cancer, sickle cell disease) [2].

Globally, more than one billion people, or around 15%, are recorded to have some sort of disability or special need. Approximately 93 million young people between the ages of 0 and 14 years live with moderate or severe requirements, and 13 million young people suffer severe burdens. According to the National Sample Survey Organization, 18.49 million people with disabilities comprise about 1.8% of the total population in India. Moreover, one-third of the total child population, which is nearly 6-10%, are born disabled in the country [3]. The overall prevalence of disability in India, based on secondary data analysis of the NFHS-5 survey (2019-21), was 4.52% [4].

The oral cavity is an indicator of the systemic function of the whole body. The oral health of children with SHCN often deteriorates more rapidly than that of their counterparts in the general population. In a systematic review conducted in 2010 by researchers Anders and Davis, it was found that the prevalence of tooth decay and periodontal disease is significantly higher in children with special needs compared to the general population [5]. Poor dental health can lead to reduced nutritional intake, impaired social interactions, difficulty in performing day-to-day activities, and associated anxiety. The AAPD recommends that preventing the risk of developing oral disease is a fundamental part of global oral health care for children with special needs. However, there are many barriers to preventive measures, including lack of accessibility to dental care, lack of understanding among dental professionals on how to care for children with SHCNs, lack of compliance with dental appointments, oral aversions, other overriding medical needs, and the financial and psychological burden on the child’s family. Dental management for individuals with special needs is planned with the aim of: (a) pain alleviation and infection control, (b) treatment or eradication of existing untreated diseases, and (c) planning for follow-up preventive measures [6].

The case series includes various preventive and therapeutic care provided to some of the challenging special needs children using non-pharmacological behavior guidance methods instead of pharmacological ones, which were easily accepted by the parents and caregivers. The primary aim of this case series is to demonstrate that preventive care for children with SHCN can improve their quality of life and make therapeutic procedures easier to plan. Although this protocol is time-consuming at the chairside, it can help develop self-esteem and confidence in these children.

## Case presentation

Case 1: Oral management of a child with attention-deficit hyperactivity disorder (ADHD) and autism spectrum disorder (ASD)

**Figure 1:**
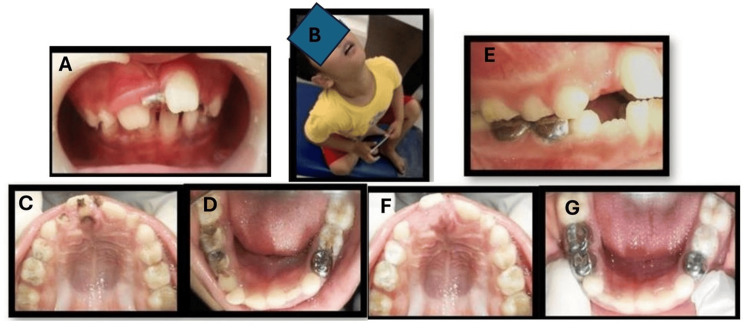
Oral management of a child with attention-deficit hyperactivity disorder (ADHD) and autism spectrum disorder (ASD) Preoperative intraoral pictures (A,C,D). Extra oral picture showing hyperactivity (B). Postoperative intra oral pictures (E-G).

An eight-year-old boy came to the Department of Pediatric and Preventive Dentistry with the chief complaint of multiple tooth decay in the lower left and right back and upper front teeth regions over the past five to six months. No history of pain was reported. The medical history revealed Level 1 autism spectrum disorder (ASD) with attention-deficit hyperactivity disorder (ADHD) since the age of five years (Figure 1B). He showed lack of attention, hyperactivity, limited social interaction, and repetitive and restricted behavior. Due to his limited attention span, short and well-organized appointments were planned.

During the first dental visit, the child was allowed to explore the dental room, which resulted in a limited oral examination. Diet counseling was provided, recommending a gluten-free and casein-free diet. During subsequent visits, behavior management methods such as tell-show-do and positive reinforcement were used. A sensory-adapted dental environment was created, involving ambient lights and rhythmic music to elicit positive behavior. A thorough intraoral examination revealed deep carious lesions in teeth 74, 75, 84, and 85 (Figure 1A,C,D). Intraoral radiographs showed radiolucency involving only enamel and dentine without pulp involvement. Therefore, Hall’s technique was performed for teeth 74, 84, and 85 due to multi-surface carious lesions, and restoration with glass ionomer cement (Type IX) was done for teeth 75 and 83 (Figure 1E,G). Extractions of root stumps of teeth 51, 52, and 61 were performed under 2% adrenaline (1:200000) local anesthesia aseptically, as their permanent successors had already erupted (Figure 1F).

During further subsequent visits, a plaque-disclosing agent was applied to all teeth surfaces showing plaque accumulation, followed by oral prophylaxis and fluoride application. Pit and fissure sealants (Clinpro® (3M, St Paul, MN, USA)) were applied to the first permanent molars. Brushing and flossing techniques were demonstrated to both the child and parents. Preventive therapy by MI Recaldent™ milk protein varnish (GC International AG, Tokyo, Japan) application was done professionally every three to six months for one year.

Case 2:Preventive oral management of child with early childhood caries with speech and hearing loss

**Figure 2:**
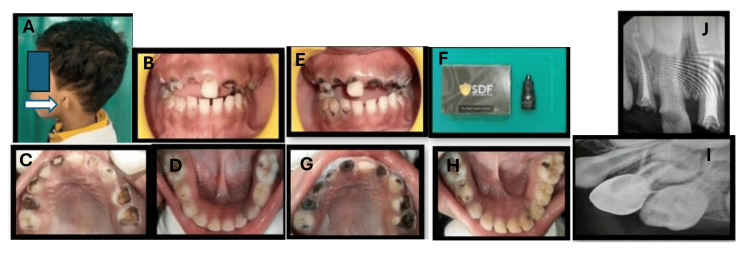
Preventive oral management of child with early childhood caries with speech and hearing loss Extra oral photograph showed Aural atresia (white arrow) (A). Preoperative photographs showing severe early childhood caries (B-D). Intraoral photographs after application of silver diamine fluoride (SDF) solution (E-H). Radiographs showing radio opaque intracanal medicament in 52, 61, 64 (I,J) followed by stainless steel crown in 64 (I).

A five-year-old boy came to the Department of Pediatric and Preventive Dentistry with a complaint of multiple decayed teeth in the upper front teeth region over the past year. There was no associated history of pain. The medical history revealed Grade 3 microtia congenitally associated with aural atresia, referred to as peanut ear (Figure 2A) as per Marx classification [7]. The child was fearful and uncooperative towards the dental team, making communication challenging. Therefore, sign language and modeling techniques were used. Wearing a face mask was avoided to allow the child to read the dentist's lips during communication. Transparent masks can also be used.

Intraoral examination revealed arrested lesions in all quadrants except for teeth 52, 61, and 64, where clinical pulp exposure was present. Since the parents were not keen on extensive procedures, chairside preventive treatment was primarily elected. In subsequent appointments, the treatment included the application of 38% silver diamine fluoride (SDF) antibacterial liquid (e-SDF® (Kids-e-Dental, Mumbai, India)) to arrest the progression of the lesions post-polishing (Figure 2E-H). Later, pulpectomy was performed on teeth 52, 61, and 64 (Figure 2I,J), followed by restoration with glass ionomer cement ESPE® (3M, St Paul, MN, USA)) in subsequent visits. Visual aids, such as video clips, were used to demonstrate tooth brushing and flossing (braided waxed) techniques. A recall visit every three to six months was planned for one year.

Case 3: Preventive management of child with early childhood caries with social anxiety disorder

A five-year-old boy came to the Department of Pediatric and Preventive Dentistry with a complaint of pain in the lower left back teeth region over the past six to seven days. The child's personal history revealed separated parents, a working mother, an abusive caretaker in the early days of his life, poor appetite, and anxiety nausea. This personal history made the case challenging.

**Figure 3:**
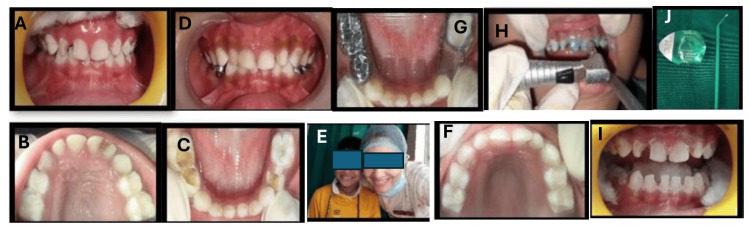
Preventive management of child with early childhood caries with social anxiety disorder. Preoperative photographs showing severe early childhood caries (S-ECC) (A-C). Mid treatment MI Recaldent varnish application post proxy paste polishing (H-J). Post-operative full mouth rehabilitation (FMR) (D,F,G). Self-confident child (E).

Intraoral examination revealed deep dentinal lesions with pulp exposure in teeth 74 and 75, a grossly decayed tooth 84, and other teeth affected with dentinal lesions (Figure 3A-C). The examination showed tenderness on vertical percussion and exacerbated feedback to thermal and electrical pulp sensitivity tests in teeth 74 and 75. Preoperative radiographic examination revealed radiolucency involving enamel, dentine, and pulp in teeth 74 and 75, and intra-radicular bone loss in tooth 84.

The treatment included pulpectomy under 2% adrenaline (1:200000) local anesthesia followed by stainless steel crowns for teeth 74 and 75, extraction of tooth 84, and placement of a band and loop space maintainer (band material: Metro^®^ (Metro, Hyderabad, India) and loop material: 0.9 mm Leowire® (Leone, Sesto Fiorentino, Italy)) (Figure 3G). Composite (ESPE® (3M, St Paul, MN, USA)) build-up was done for teeth 51, 52, 53, 61, 62, 63, 71, 72, 81, and 82 (Figure 3D,F,G). Preventive therapy involved the application of MI Recaldent™ milk protein varnish (GC International AG) at zero, three, six, and 12 months follow-up, post-polishing to remove plaque and deposits (Figure 3H-J). Proper behavior shaping is required to manage a special needs child, resulting in a happy child (Figure 3E).

Case 4:Single visit feeding obturator for an infant with cleft palate and cleft lip

**Figure 4:**
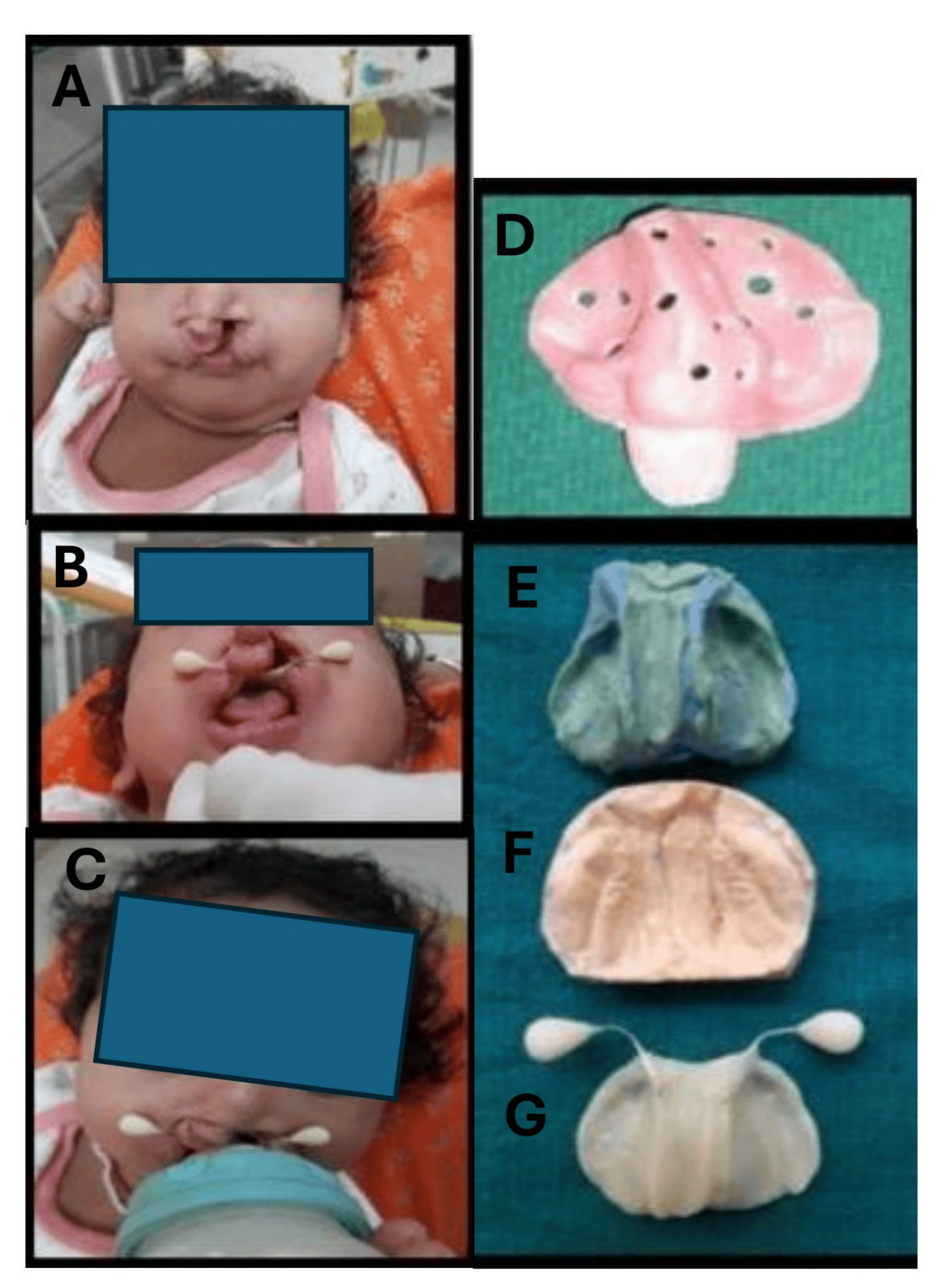
Single visit feeding obturator for an infant with cleft palate and cleft lip. Preoperative photograph: Veau class VI (A). Postoperative photographs showing retentive feeding plate with bottle training (B,C). Custom tray fabricated on the primary impression made by putty silicone material (D). Secondary impression registered with putty and light body silicone material on which master cast was fabricated with type 4 die stone and final feeding plate (E-G).

A three-month-old boy came to the Department of Pediatric and Preventive Dentistry with a complaint of difficulty in feeding since birth. There was no history of craniofacial clefts in the family of the child. The pregnancy of the mother was uneventful, and there was no history of previous treatment or surgery for the defect. Intraoral examination revealed non-syndromic bilateral cleft lip and palate (Veau Class IV) [8] (Figure 4A - preoperative and Figure 4B - postoperative). Preventive treatment included the fabrication of a feeding plate (Figure 4D-G).

The impression was made when the infant was fully awake in a semi-upright position in the mother's lap, and no pre-medication was given. A primary tray was selected based on the size of the infant's oral cavity. The preliminary impression was recorded using silicone-based material (putty consistency) (Waldent® (Waldent Innovations, New Delhi, India)) on the primary tray (Figure 4D). Beading and boxing were done with modelling wax (Prime® (Prime Dental Products, Thane, India)), and a cast was fabricated (Figure 4E,F). Upon the primary cast, a secondary/custom tray was fabricated using cold cure acrylic material. A handle was created in the anterior part of the tray to provide support while registering the impression. The custom tray was trimmed and polished to fit into the infant's oral cavity. Holes were created on the tray to allow excess impression material to easily escape. Silicone-based material, initially with putty consistency followed by light body consistency, was used for recording the secondary impression. The secondary cast was fabricated using type 4 die stone (Neelkanth® (Neelkanth Ortho Dent, Jodhpur, India)). The final obturator was prepared using cold cure acrylic material (Figure 4G) as it was to be delivered the same day. Retention was checked, and training was provided to the mother and child (Figure 4B,C). Instructions regarding cleaning and storage of the obturator were given to the parents. Follow-up was done every three months.

## Discussion

Children with SHCN have relatively poor oral hygiene, which results in an increased prevalence of dental caries, gingival, and periodontal diseases. Due to the burden of the child's medical health, parents usually do not seek dental care, and their dental needs are unmet. Hence, in 2008, special care dentistry was established with the aim of providing preventive and therapeutic treatment for people who are incapable of accepting routine oral care services due to their limited physical, mental, sensory, cognitive, behavioral, or emotional development [9]. Children with SHCN fall under the high caries risk category, which further threatens their overall health [10]. Essential comprehensive oral health care for children with SHCN is recommended as a preventive strategy by AAPD.

This case series focuses more on the preventive aspect of treatment. Among various non-pharmacological behavior guidance techniques, tell-show-do (TSD) and positive reinforcement are widely used by pediatric dentists and have rarely encountered any reluctance or refusal by patients and parents. Sensory-adapted dental environments (SADE) are an acceptable technique to reduce anxiety in patients with SHCN [11].

Parents' and caregivers' training and education on diet counseling are of utmost importance for ensuring regular and proper oral hygiene protocols for the child. Limiting the consumption of sugars in foods and beverages and maintaining a healthy dietary pattern will reduce the risk of dental caries at an early age. Establishing an early brushing of the teeth habit will result in better oral hygiene. For children with limited adaptive functioning, parental guidance is important to guarantee an efficient and safe brushing of the teeth habit. The treatment of choice for children with caries includes professional topical fluoride application every three months.

Children with hearing loss face challenges when using conscious sedation because they cannot provide verbal feedback, which is a key component of the definition of conscious sedation. Inhibiting the progression of dental caries in these children is essential [12]. A 38% SDF containing 5% fluoride assists in arresting carious lesions through the action of fluoride ions that remineralize the enamel and dentin, and silver ions that have an antimicrobial effect on the treated carious dentin [13]. SDF retains approximately two to three times more fluoride than sodium fluoride, stannous fluoride, or acidulated phosphate fluoride found in varnishes, gels, or foams [14]. Prior informed consent is required due to the expected staining of treated cavitated lesions when using SDF. SDF is considered to be effective and well-accepted by parents and dentists for arresting caries progression in pediatric dentistry [15].

Children with ADHD and ASD are generally on some medications like Dexedrine (dexamphetamine) or Ritalin (methylphenidate hydrochloride), which lead to a high prevalence of dental caries. Therefore, fluoride as a preventive measure is very beneficial to them [16]. MI varnish with Recaldent contains 5% sodium fluoride with CPP-ACP (casein-phosphopeptide-amorphous calcium phosphate), which enhances enamel acid resistance. The presence of fluoride in the varnish leads to the formation of fluoridated crystals (fluoroapatite or hydroxyfluorapatite) that are difficult to dissolve and easy to repair. To stabilize the fluoride, calcium and phosphate ions are added to varnishes. The stabilization of calcium and phosphate by casein phosphoprotein binds to amorphous calcium phosphate and prevents the growth of calcium and phosphate ions to a critical size for nucleation and phase transformation [17].

Non-restorative cavity control (NRCC) can be an alternative treatment option where the cavity is made accessible by removing the overhanging enamel or slicing the surface in the case of a proximal cavity [18]. Cooperation of the parents, caregiver, or patient is a prerequisite for successfully implementing NRCC. According to care pathways for caries management, the child was recalled at three, six, and 12 months, with an X-ray at six months [19]. Active surveillance or periodic monitoring of non-cavitated white spot lesions and caries progression is required during follow-up visits.

In cleft patient cases, surgical closure is usually planned between nine and 18 months of age. Various feeding techniques are recommended for infants with CLP.

A neonatal feeding obturator helps create a seal between the oral and nasal cavities, aids in nutritional gain, ensures proper tongue position, prevents nasal regurgitation, and allows the generation of intraoral pressure [20]. When recording impressions in infants with CLP, it should be done carefully to avoid respiratory obstruction and cyanotic episodes. Proper oral hygiene measures are essential to prevent fungal growth on the palatal surface during the use of the obturator.

The primary goal of dental care is to offer anticipatory guidance that prevents dental caries and reduces the need for hospitalization. It is particularly important to avoid general anesthesia for children with SHCN as there may be potential underlying health conditions and sometimes it is difficult for them to cope up with hospital-based care. By identifying the need for treatment through preventive evaluations and ensuring its availability, we can prevent more severe systemic issues [10].

## Conclusions

Dental clinicians must adopt preventive strategies and individualized treatment plans to manage caries effectively and enhance the comfort levels of these children. By understanding the psychological and social challenges faced by children with SHCN, dental teams can provide comprehensive care that improves their quality of life and supports their growth, function, and appearance.

## References

[1] Definition of special health care needs, The American Academy of Pediatric Dentistry. The Reference Manual of Pediatric Dentistry.

[2] Estrella MR, Boynton JR (2010). General dentistry's role in the care for children with special needs: a review. Gen Dent.

[3] (2023). Disabled Persons in India, Report No. 485. https://mospi.gov.in/sites/default/files/publication_reports/485_final.pdf.

[4] Pattnaik S, Murmu J, Agrawal R, Rehman T, Kanungo S, Pati S (2023). Prevalence, pattern and determinants of disabilities in India: insights from NFHS-5 (2019-21). Front Public Health.

[5] Anders PL, Davis EL (2010). Oral health of patients with intellectual disabilities: a systematic review. Spec Care Dentist.

[6] Dean JA (2022). McDonald and Avery’s Dentistry for the Child and Adolescent, 11th edition. http://evolve.elsevier.com/cs/product/9780323698207?role=student.

[7] Andrews J, Kopacz AA, Hohman MH (2024). Ear microtia. StatPearls.

[8] Veau V (1932). Division palatine: anatomie, chirurgie, phonétique. Paris: Masson et Cie.

[9] Nic Iomhair A, John M (2020). Facilitating patient-centred care for special care dentistry patients: a quality improvement project in the community dental service. BDJ Open.

[10] American Academy of Pediatric Dentistry (2024). Management of dental patients with special health care needs. The Reference Manual of Pediatric Dentistry.

[11] Dhar V, Gosnell E, Jayaraman J (2023). Nonpharmacological behavior guidance for the pediatric dental patient. Pediatr Dent.

[12] Standards for Conscious Sedation in the Provision of Dental Care and Accreditation. Dental Care (v1.

[13] Crystal YO, Niederman R (2019). Evidence-based dentistry update on silver diamine fluoride. Dent Clin North Am.

[14] Shah S, Bhaskar V, Venkataraghavan K, Choudhary P, Ganesh M, Trivedi K (2013). Efficacy of silver diamine fluoride as an antibacterial as well as antiplaque agent compared to fluoride varnish and acidulated phosphate fluoride gel: an in vivo study. Indian J Dent Res.

[15] Abdulrahim R, Splieth CH, Mourad MS, Vielhauer A, Khole MR, Santamaría RM (2023). Silver diamine fluoride renaissance in paediatric dentistry: a 24-month retrospective and cross-sectional analysis. Medicina (Kaunas).

[16] Salam TAA, Ummer M, Abdullah Alowairdhi A, Khalid Alsubait A, Marwan Aljuhani S, Abdullah Alzahrani A, Ali Alqahtani A (2023). Management of attention-deficit hyperactivity disorder children for dental procedures. Cureus.

[17] Cochrane NJ, Cai F, Huq NL, Burrow MF, Reynolds EC (2010). New approaches to enhanced remineralization of tooth enamel. J Dent Res.

[18] van Strijp G, van Loveren C (2018). No removal and inactivation of carious tissue: non-restorative cavity control. Monogr Oral Sci.

[19] American Academy of Pediatric Dentistry (2024). Caries-risk assessment and management for infants, children, and adolescents. The Reference Manual of Pediatric Dentistry.

[20] Jones JE, Henderson L, Avery DR (1982). Use of a feeding obturator for infants with severe cleft lip and palate. Spec Care Dentist.

